# Affective Neuroscience Contributions to the Treatment of Addiction: The Role of Social Instincts, Pleasure and SEEKING

**DOI:** 10.3389/fpsyt.2021.761744

**Published:** 2021-11-23

**Authors:** Daniela Flores Mosri

**Affiliations:** Department of Psychology, Psychoanalytic Psychotherapy, Neuropsychoanalysis, Universidad Intercontinental, Mexico City, Mexico

**Keywords:** affective neuroscience, addiction, treatment, SEEKING system, social instincts, pleasure, neuropsychoanalysis

## Abstract

Addiction is an illness prevalent in the worldwide population that entails multiple health risks. Because of the nature of addictive disorders, users of drugs seldom look for treatment and when they do, availability can be difficult to access. Permanence in treatment and its outcomes vary from case to case. Most models work from a multidisciplinary approach that tackles several dimensions of addictive disorders. However, the different etiological factors claim for a personalized treatment to enhance opportunities for better results. Problems in relationships with others play an important role in the etiology and the recovery process of addiction. This paper focuses on the social-environmental causes of addiction based on an affective neuroscience approach that attempts to integrate the interplay between social instincts, pleasure, and the SEEKING system in addiction. To advance toward better treatment strategies, it is pertinent to understand the limitations of the current multidisciplinary models. Acknowledging the social nature of the human brain may help to identify the quality of different types of traumatic early life experiences in drug users and how to address them in what may become a neuropsychoanalytic treatment of addiction.

## Introduction

Addiction is a complex and prevalent disorder worldwide (1, 2). The extent of the individual and social related consequences of drug use is costly and harmful in various ways. An estimated of 269 million people used drugs during 2017 and 35.6 million suffer from a drug use disorder (1). Prevention and treatment do not seem to cover the scope of this unfathomable problem, despite major efforts to address it. One in eight people receives treatment each year (1). An attempt to explain the failure and/or insufficient results of the existing models of treatment may lead to suggestions to improve the available clinical approaches. Further investigation of the causes and characteristics of addiction seems necessary, as it is one of the major health concerns in many countries. The treatment of addiction is commonly multidisciplinary, e.g., it covers neurobiological, psychiatric, social, and psychological aspects. However, the general outcome of the treatment is modest and short-lasting (3). Recent neurobiological advances have contributed to an improved understanding of the mechanisms of addiction, leading to the development of different medications to diminish drug use or to achieve abstinence (4). Cognitive-behavioral strategies are effective in controlling craving and promote abstinence. Social support has been recognized as an important aspect of the treatment of addiction (5–7). Most users experienced life backgrounds that challenge social cohesion and lack positive relationships. Such circumstances are stored in different memory systems with an impact on affect regulation, which contributes to a neurochemical hypothesis to explain an enhanced vulnerability to use drugs. The differentiation of pleasure, reward, and SEEKING seems necessary to expand on the modalities of treatment currently available. Acknowledging that a person who suffers from an addictive illness is a special case of a neurological patient may add a frequently forgotten dimension to a self-locking disorder that requires personalized strategies for treatment. The current paper highlights the need for addressing the subjective experience of living with addiction which has not been the main focus of previous studies. Neurobiological aspects, genetic vulnerabilities and early life experiences all contribute to the onset of an addictive disorder influenced by what the person subjectively feels. The correlates of traumatic separation distress and its effect on other social instincts are addressed as important contributors to the vulnerability to addiction. Considering the subjective experience of unmet social needs may add to the current models of treatment.

This paper takes an integrative perspective that considers the various dimensions of addiction. It does not intend to elaborate on every dimension, but to hypothesize their interactions by emphasizing the relevance of early social trauma. The nature of addiction is first explored as a self-locking disorder to highlight the inherent difficulties that addiction poses for its treatment. The influence of social instincts on addiction is later explored by briefly reviewing the four social instincts as proposed in Panksepp's affective neuroscience to hypothesize their role at the onset of addiction as separation-distress trauma. Next, the differentiation between SEEKING, pleasure, and reward is addressed to elaborate on the subjective aspects of addiction. Last, an integration of several dimensions involved with addiction is attempted to discuss potential contributions to the current models of treatment from a social perspective. A neuropsychoanalytic viewpoint is proposed as an integrative approach to analyze individual cases in their several dimensions, such as affect and procedural-emotional memories. A frame of reference that integrates data from different fields offers new insights into addiction and its treatment.

### Addiction as a Self-Locking Disorder: Challenges for the Treatment

**Figure 1:**
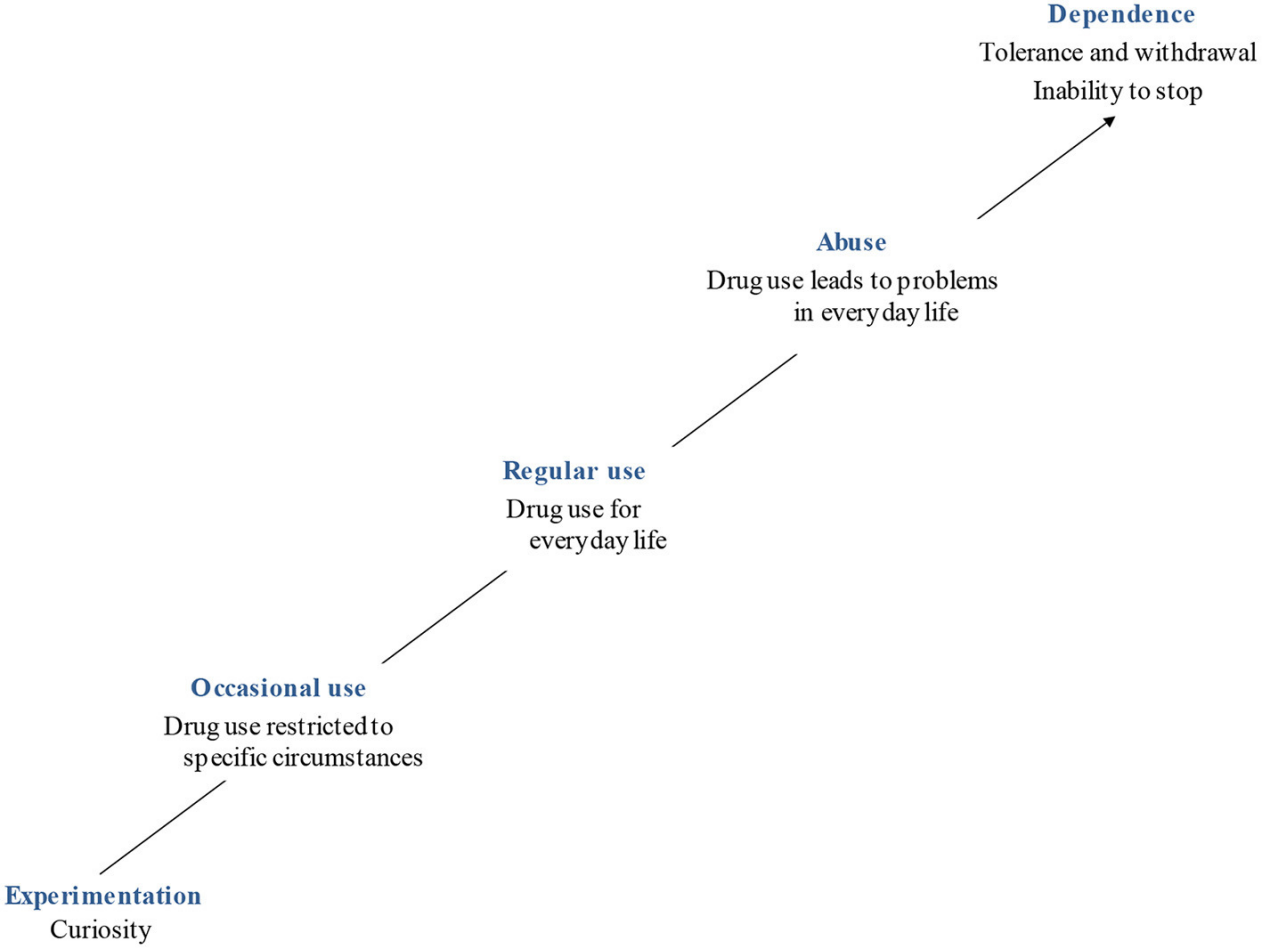
From drug use to the risk of addiction.

The main models of treatment for addiction take a multidisciplinary approach that addresses several dimensions of this complex disorder. The treatment may entail detoxification, pharmacological agents (4, 8), and psychotherapy, which may include individual, group, and family approaches (9, 10). However, the most challenging impediment to treat addiction is that users seldom ask for help. People who use drugs frequently report experiencing a pleasurable or helpful effect despite the declining results as tolerance increases. Hence, repeated interventions may be necessary before the person agrees to be treated. It may also take several attempts before the treatment succeeds. It seems unlikely that the current models of treatment ignore any of the different dimensions of addiction. Nonetheless, the question of how to make the different approaches more efficient remains. When all or most aspects of addiction are addressed, alternatives to improve the outcomes of different treatments narrow to looking into more detail at some causes and course of the disease. Addiction can be described as a self-locking illness. A person usually tries drugs, reporting that they were curious to know what it felt like to be intoxicated. If they decide to use drugs subsequently, the risks of developing an addictive disorder increase. Trying different drugs and/or using a group of them regularly may become habitual for many users who do not foresee potential abuse and dependence problems (Figure 1). The gradual progression toward addiction impedes many users from noticing that they cannot stop using the drug. At this stage, tolerance, craving, and withdrawal are experienced. Nevertheless, many users still consider that they can control their drug use (10–13). Thinking that they do not have a problem, i.e., denial, accounts for one of the key characteristics of addictive disorders and it explains why users seldom look for help.

It is sometimes considered that users are unaware of the negative consequences of using drugs. This is rarely the case (7, 9, 14). In fact, it has been suggested that in order to use drugs, the person must resort to dissociative defense mechanisms that facilitate that they behave as though their mind could live separate from their body and that using drugs entails no potential harm (10). Users may also rationalize the negative consequences to allow themselves to keep using drugs. As a result of recruiting several defense mechanisms, some users consider the harmful effects of drugs as less important than they are; some may feel that they can control both the use and the damaging effects; others may report that the harmful effects of drugs are irrelevant as they are aware of their inevitable progression toward death regardless of the use of drugs. One common belief amongst users is that if they have knowledge about the drug, then they will use it without consequences, as they can manage the negative outcomes of consumption (15). Denial, dissociation, omnipotent control, and rationalization are some of the defensive mechanisms that diminish the awareness of having a problem. Treatment is then not considered. The disease is essentially inaccessible, namely, self-locking. The use of defenses may entail neurobiological correlates operating in addiction including the modifications of the dopamine mesolimbic pathway and impaired functioning of the prefrontal cortex (PFC), which may contribute to the scarce insight related to the recognition of addiction (7, 8) and to the compulsory use of the drug (13). Behavioral, cognitive, biological, and subjective aspects are coherent in the presentation of addiction.

In sum, the person uses drugs despite their negative consequences because they expect to control them; the latter includes the risk of developing an addictive disorder, namely, no person starts using drugs intending to develop addiction. However, there are important neurobiological implications that contribute to explain the use of the defenses described before. When addictive drugs recruit the mesocortical limbic dopamine pathway, they hijack a system involved in motivation, incentive salience (16, 17) and executive functions (3, 8, 12) amongst others. Olds and Milner (18) named it the reward system as they believed they had found the pleasure centers of the brain. Years later, Panksepp (19) challenged Olds and Milner's viewpoint by expanding the study of the circuit to a SEEKING system that involves an urge to explore the world and to predict potential rewards (17). The SEEKING system looks for rewards, but it does not entail the reward. The implications of this system in addiction partially clarify the type of thinking that characterizes addiction, contributing to its self-locking characteristics. Panksepp (19) has argued that an increased activity of the SEEKING system results in distorted thinking. Overarousal of SEEKING relates to superstitious ideas that establish false associations between certain stimuli and the anticipation of a reward. The false association attempts to predict a causal relationship despite the repeated absence of a reward. In brief, the overarousal of the SEEKING system may contribute to the modified “superstitious” thinking that expresses some paradoxes of addiction (20), e.g., using addictive drugs to survive while this behavior leads to harmful effects, sometimes even to death. This type of thinking reduces the chances that users notice their false associations, thus enhancing the risk of addiction while simultaneously decreasing the chances of looking for help. Moreover, Ceceli et al. (8) have recently reviewed the dysfunction of the PFC in addiction. They emphasize the impaired response inhibition and salience attribution (I-RISA) model as proposed by (21) to explain the difficulties in suppressing disadvantageous behaviors related to overvaluing drug reinforcers and undervaluing reinforcers unrelated to drug use. Using addictive drugs results in impaired prefrontal functions such as self-control and insight into illness. The latter components all contribute to the self-locking nature of addiction.

The self-locking characteristic of addiction also leads to distorted goals and beliefs in users who accept treatment. They may agree to be treated expecting to continue drug use while avoiding the harmful consequences, including neurobiological and psychological dependence (10). This belief expresses the need for the addictive substances and reluctance to engage with the treatment, frequently leading to relapse, failure and/or disappointment. Once the dependence stage is active, it becomes increasingly challenging to break the self-locking essence of addiction. Thus, focusing on a deeper understanding of the causes of addiction may be valuable. The genetic, developmental, and social factors are to be considered. An account of the complex social aspects follows.

### The Influence of Social Instincts in Addiction

The social aspects related to addiction can be addressed from a cultural viewpoint. The need for acceptance by peers and cultural agreements to use legal and illegal drugs stand out as concurrent causes of addiction (22). However, the primary process instinctual foundations of the human need for others are crucial to the understanding of addiction. Human beings depend on others to survive. When primary relationships are characterized by failure to provide a secure base, other social instincts may be compromised, which results in an enhanced vulnerability to psychopathology, including addiction (7, 23).

Because of the state of immaturity at the time of birth, mammalian organisms require a primary caregiver while they are young. The need for caregivers is also expressed in situations in which an organism cannot meet their needs on their own, e.g., during illness. The latter implies a social brain that emphasizes the necessity of others to survive. We depend on one another throughout life; infancy is the time in which more care from others is required, particularly from the mother. Separation from the caregiver can cause significant feelings of loneliness and sadness (19, 23) that quickly subside when the reunion with the caregiver takes place. If separation distress persists, it can become a traumatic experience that may influence other types of relationships.

Separation distress has been linked to the endogenous opioid system (EOS) (24–26). Mu opioid receptor (MOR) activity may be persistently low while kappa opioid receptor (KOR) activity may be high, which translates into feelings of PANIC/GRIEF in Pankseppian affective neuroscience, as a resource to express the need of a primary caregiver when in situations of helplessness (19, 23). The protracted activation of the PANIC/GRIEF system can translate into an enhanced vulnerability to addiction (20). If one important cause of addiction relates to traumatic separation distress, it is possible to hypothesize that drug users may resort to opioid drugs as an attempt to regulate the feelings derived from protracted PANIC/GRIEF. However, many people use substances that primarily recruit neurochemical pathways not directly related to the endogenous opioid system (EOS). Three main hypotheses are suggested to explain such an inclination (1). Most addictive drugs target the dopamine mesocortical-mesolimbic pathways, which in turn may activate MORs in the nucleus accumbens, then resulting in a temporary relief of PANIC/GRIEF feelings (2). Drugs that do not target the endogenous opioid system directly may be used to relate to people, e.g., in recreational activities such as social gatherings which may increase MOR activity (3). Drugs that target the mesocortical-mesolimbic dopamine pathway may facilitate a subjective feeling of general positive expectation and hope, including that of relatedness with others during intoxication.

The enhanced dopamine activity of the SEEKING system that results from the use of addictive drugs generates feelings of euphoria, while hedonic experiences relate to endogenous opioid activity (17, 23). Addiction becomes a key source of frustration, as no satiety is found when there is a reduced sensitivity to alternative incentives that help to solve problems and to satisfy needs. If problems and needs remain unsolved, no pleasure can be experienced, including that derived from spending quality time with friends and family. A multidisciplinary approach to treat addiction must consider the role of unmet social needs as a relevant contribution to explain the onset of compulsive drug use. As one of the frequent social difficulties present in drug user's lives, enhanced FEAR may impede relating to others as part of renewed traumatic separation distress. Different modalities of traumatic separation distress may explain the need to stimulate the dopamine mesocortical-mesolimbic system related to a depressive cascade that includes EOS dysregulation derived from prolonged separation distress experiences (25).

#### The Social Nature of the Human Brain^1^

According to (19, 27), the mammalian brain is pre-wired with instincts to survive. When exposed to an unconditioned stimulus, a specific behavioral pattern (an unconditioned response) is ready to solve problems in the world that are signaled by an affective subjective experience. Unpleasant affects drive the organism to act toward resolution of conflict. As instinctual patterns will be insufficient in most circumstances, primary-process emotional responses learn from experience by associating cues in different memory systems; this is the secondary-process level, followed by tertiary cognitions that recruit higher cortical regions to supplement the brain's problem-solving abilities. Mammalian organisms need to relate to others in order to survive. This means that as human beings, we have a social brain with instinctual tendencies to SEEK for relationships. Panksepp's affective neuroscience (19) has proposed the existence of four pro-social affective instincts in the mammalian brain, (1) the LUST system, (2) the CARE system, (3) the PANIC/GRIEF system, and (4) the PLAY system. Hence, there are four formats in which relationships can be established that entail different affective qualities. Instincts enhance our chances of survival. The existence of different types of social relationships implies that they satisfy different needs. A brief description of each pro-social system follows.

An instinct to reproduce links to the feeling of sexual attraction. Panksepp (19, 23) proposed that the LUST system favors the survival of the species through the subjective experience of sexual affects. Regulated by different hormones in males and females, the activation of this circuit favors sexual drive and romantic love at a secondary and tertiary-process level when in combination with other emotions, e.g., CARE. One potential result of sexual interaction is reproduction. Mammalian organisms are born immaturely and require the CARE of adult organisms that will meet the needs of babies. The CARE system protects the survival of others and drives toward looking after those in need. CARE is present in both males and females, albeit it is more active in females because of hormonal mediation. CARE thus promotes social interaction to provide safety and comfort to others in helpless situations that extend beyond babies to ill and old people, for example.

The accompanying pair of CARE is a separation distress system (26) or PANIC/GRIEF system, which is more active in young and immature organisms. Panksepp (19, 23) linked PANIC with the attachment system described by (28, 29) in which the need for CARE makes the young ones feel separation distress when their primary caregiver is away. PANIC/GRIEF contributes to the establishment of the first type of relationship in life; it is subjectively described as the need to be taken care of. The state of immaturity inherent to young mammalian organisms will require the transit through several developmental stages whose success depends on a primary caregiver, frequently but not exclusively, the mother. When the young one and the caregiver experience separation, feelings of PANIC arise in the young one who produces separation-evoked distress vocalizations, crying in humans. These cries or vocalizations are part of a protest phase to call the primary caregiver with the goal of reuniting. If the caregiver and the young one get back together, the PANIC reaction ends. However, if the separation lingers, the protest phase is followed by a despair phase in which the young organism retracts and stops the distress vocalizations or crying. The latter is an attempt to avoid getting attention of dangerous others and to save energy as it becomes unknown whether the primary caregiver will indeed return. In brief, the mechanisms of the PANIC/GRIEF system favor attachment to those who provide CARE. This early relationship between a primary caregiver and a young organism favors survival of the young ones, which explains the subjective feeling of psychic pain (23–26) during separation. The young organism will try to avoid this negative affective experience by staying close to their primary caregiver. Other feelings that present when the PANIC/GRIEF system is active are loneliness and sadness. In sum, the younger the organism, the more they need bonding with others to survive, which is why there is a need for attachment to a primary caregiver.

The last pro-social system proposed by (19, 23) is PLAY. Its activation promotes vigorous physical activity amongst peers that produces laughter. The associated subjective experience is that of joy and having fun while physically interacting with others. Frequent related behaviors may include persecutions and tickling. Panksepp (19) hypothesized that this system may promote the establishment of social hierarchies, social cooperation and exercising of emotional, cognitive, and behavioral patterns as it favors learning social rules linked to cooperation and competition. This pro-social instinct may, in turn, foster friendship and strategies to avoid social rejection. Solms (30, 31) has proposed that it may play a role in empathy by comprehension of the 60/40 rule, 70/30 according to Panksepp (19, 23, 32) in which participants at PLAY should take turns to win and lose for this activity to keep its fun nature. Relations amongst peers may promote group activity for problem solution.

**Figure 2:**
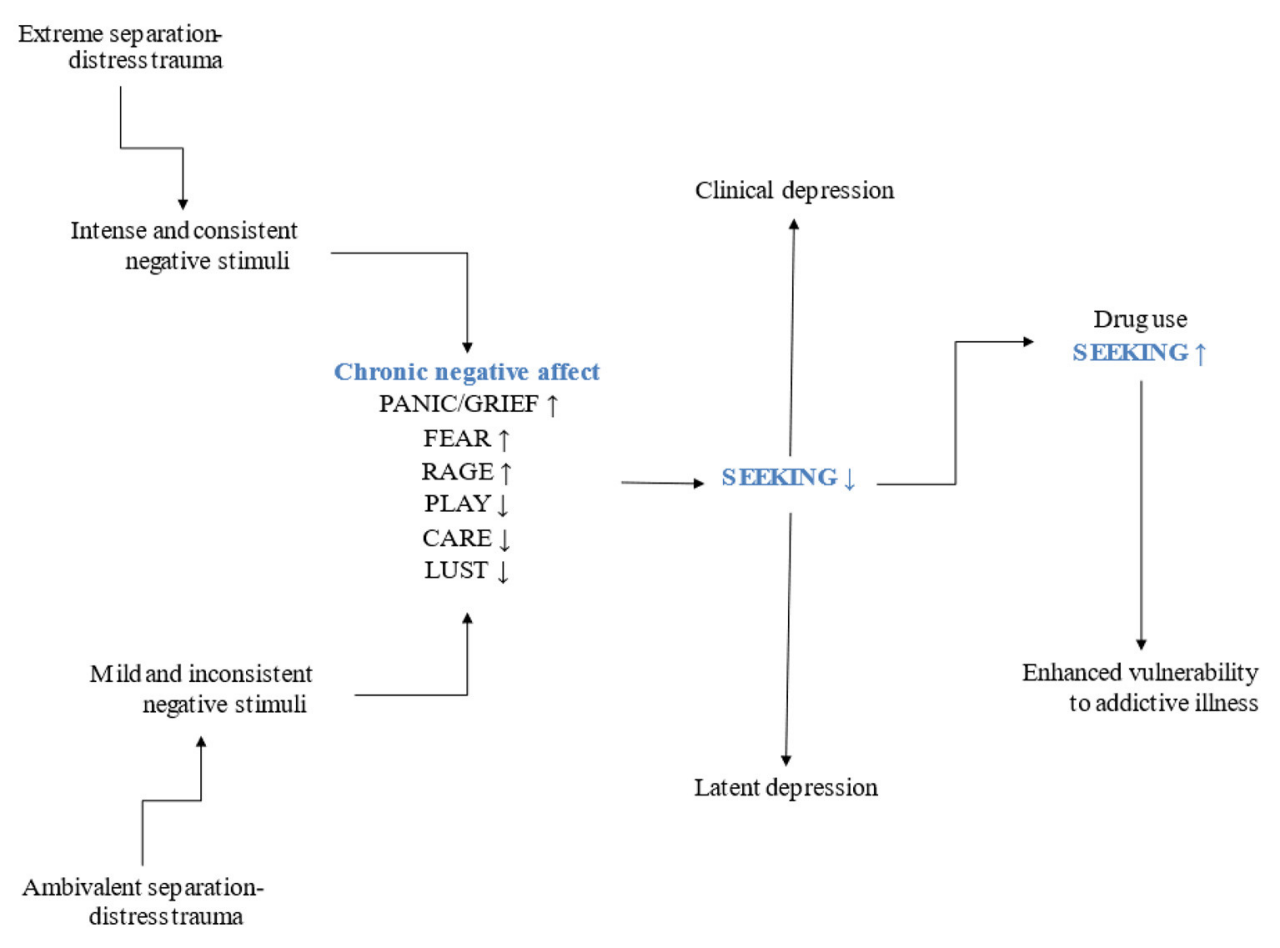
The two types of separation-distress trauma and their impact on affect.

**Figure 3:**
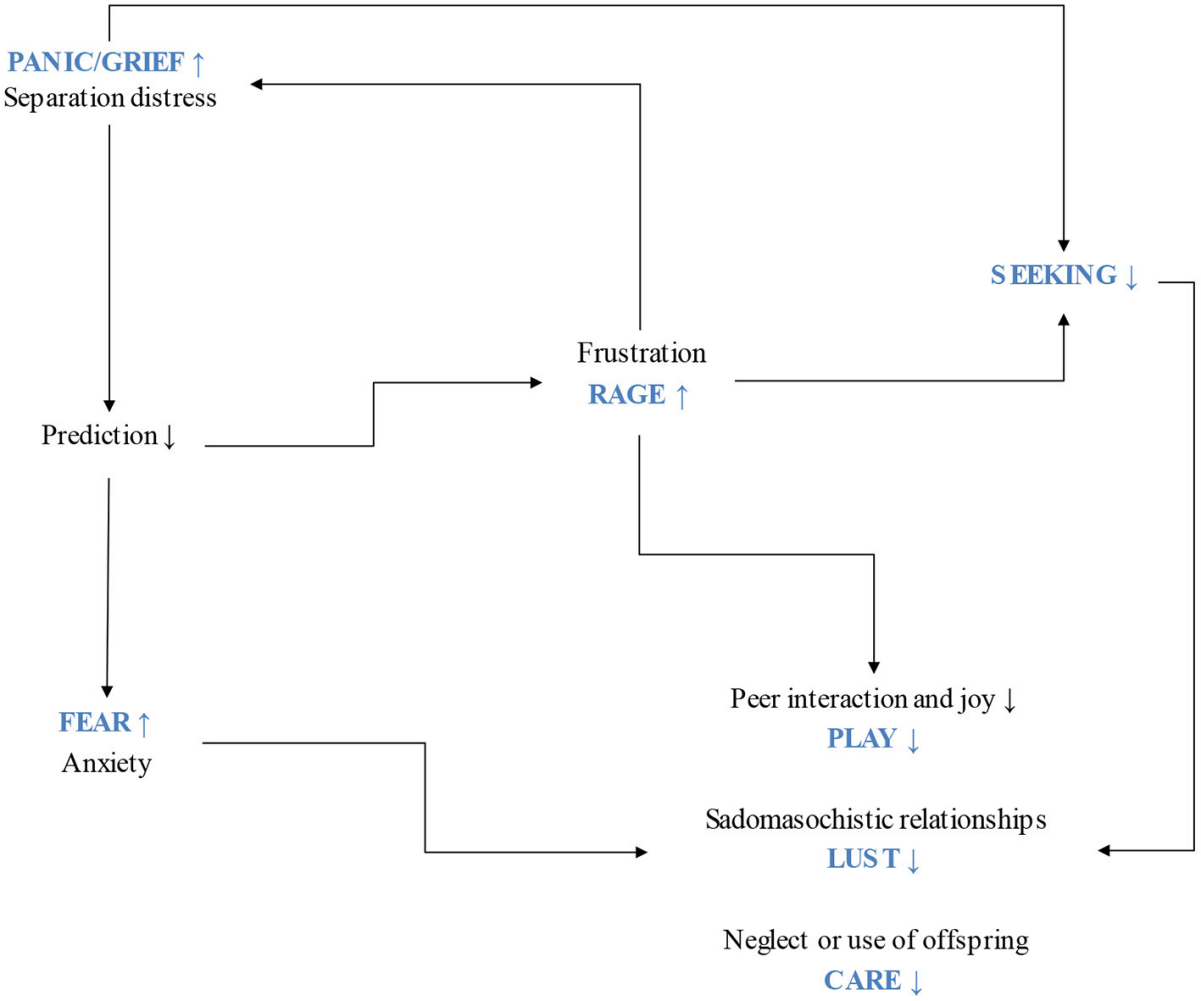
Protracted separation distress affects the basic emotion systems.

Other basic emotion systems in Panksepp's affective neuroscience (19) include the FEAR and RAGE systems, which prepare the organism to react against stimuli that may endanger survival. FEAR uses flight and/or freezing behaviors while RAGE takes fight as a response to perceived risk. These two systems may contribute to a pro-social approach under certain circumstances, for example, when FEAR and RAGE prompt defending others against different types of danger. Considering how essential social bonds are to survival, the different instances in which they fail may become a crucial aspect, resulting in one reason to use drugs. PANIC/GRIEF, FEAR and RAGE inhibit SEEKING activity (see Figures 2, 3).

#### Social Trauma: Separation Distress as an Essential Contributor to the Etiology of Addiction

It is well-known that addiction is favored by several types of vulnerabilities, including genetic, psychiatric, social, and contextual difficulties (4, 33). However, to emphasize the obvious, any of the latter requires that the person uses drugs to be at risk of developing an addictive illness. It is here proposed that deficits in social relations have a pervasive influence on neurobiological dysregulation that translates into protracted negative affective experiences. Attaching to a primary caregiver is the most fundamental relationship, as survival depends on it (19, 34, 35). It is an instinct that helps to regulate other types of social instincts and affects, as several developmental theories have asserted [e.g., (36)]. To illustrate one of these instances, Panksepp (19) clarified that PLAY emerges within a supportive and secure base. A proper understanding of pro-social affects entails considering the networks in the brain that are associated with our basic need of relating to others and their specific neurochemistry. Prolonged PANIC/GRIEF activations during infancy because of the separation of the primary caregiver can lead to psychic pain (25) and illness. Problems in this CARE-PANIC relationship may result in a general vulnerability to psychopathology, including addiction (20, 37) and may become an impediment to the satisfaction of other social instincts.

It is here proposed that the context of protracted early separation distress can take two main formats of PANIC/GRIEF activation that may become traumatic. The first relates to extreme abandonment, rejection and/or maltreatment by the primary caregivers. The second refers to ambivalent and confusing relationships with the primary caregivers. For the first type, there is an affective certainty of a constant unpleasant and painful feeling that characterizes the relationship with the primary caregivers predominantly. For the second type, the relation pattern becomes uncertain as the primary caregivers offer an inconsistent type of relationship in which they sometimes reject, abandon, or abuse their children, but in other moments, they may be caring and loving. Both cases may cause depressive symptoms and behaviors (20) that recruit a specific neurochemistry related to PANIC, namely, overactive dynorphin and KORs and low activity in MORs (25, 26). Oxytocin and prolactin are also major players involved with PANIC/GRIEF feelings (19, 23, 27) as they play an inhibitory role that can help to reduce separation distress feelings. They contribute to reinforcing social relations, including that of the primary caregiver and the infant, by enhancing attachment.

Neurochemical systems work in cascades that consequently affect other neuromodulating pathways, resulting in chronic depressive feelings that, in turn, dysregulate the functioning of the seven basic emotion systems (25) (see Figure 3). Depression underlies addictive illness (20). It is widely accepted that there is an unpleasant subjective experience that reinforces drug use during the withdrawal stage of addiction [e.g., (3, 38, 39)]. However, a negative affect before developing an addictive illness is less discussed despite the perspicuous formulations of a self-medication hypothesis (40, 41). This perspective challenges the simplistic idea that using drugs is merely for recreational purposes. It may be the case that users report such goal, nonetheless, using drugs entails harming oneself (9, 10, 42). Self-aggressive behaviors can be understood as indicators of depression (for a detailed account of the role of depression in addiction) (20, 37). Based on a psychoanalytic explanation, object loss can reflect a narcissistic type of relationship that instigates a protest reaction against parting with an object (42–44). This narcissistic relationship may derive from protracted separation distress during childhood. RAGE can result from the object frustration implied in PANIC/GRIEF activations. Aggressive feelings are then expressed against an internalized split representation of the negative affective qualities of the lost object that translate into self-aggression. Using drugs while knowing that they harm the body and the mind should be understood as a self-aggressive behavior motivated by unconscious associative patterns learned from early experience and consolidated in implicit memory structures at a secondary-process level according to Panksepp's nested BrainMind hierarchies (45). Implicit and emotional patterns hardly change after they have been learned, which may lead to a pathological attempt to mourn object loss. The unconditioned love and availability expected from the primary caregivers was not present and the person looks for an explanation in their mistakes or failed endeavors, namely, in failed ego ideal expectations. Psychoanalytically speaking, object control was not achieved, meaning that mild or intense chronic separation distress was present from the start, generating depressive feelings that necessarily relate to neurochemical modifications, featuring the endogenous opioid system (37) (see Figure 2).

The hypothesis of depression underlying addiction then relates to object loss. This loss can have an unconscious quality that affects neurobiological circuits chronically, prompting the use of defenses against the negative affects implied (46, 47). The latter explains latent and discrete presentations of depressive symptoms as opposed to clinical depression (20). The extreme context of early abandonment, rejection and/or maltreatment may relate to clinical depression more frequently, while the ambivalent context may generate latent depressions that are easy to miss in the clinical assessment. The extreme context leads to few prediction errors in terms of the certainty of experiencing negative affects when interacting with the primary caregivers. The satisfaction of different needs is not met, resulting in high free energy. On the other hand, the ambivalent and unreliable experience of separation distress trauma leads to frequent prediction errors and hence increased free energy and entropy (48). The affective consequence for both cases is the repetitive feeling of frustration of an instinct to attach, which in turn results in RAGE and in diminished activity of the SEEKING system. The combination of PANIC/GRIEF, RAGE and low SEEKING activation is characteristic of depression (20, 25). The need to modify the subjective and conscious experience of negative affects becomes imperative. The person then becomes more vulnerable to using drugs and, therefore, to addiction.

Solms (30, 49, 50) has proposed that negative affect drives behavior toward meeting unsatisfied needs. The goal is to recover homeostasis, which is signaled by the experience of pleasure. He has hypothesized that successful strategies for problem solving automatize in procedural memory structures. When attempts to meet a certain need are repeatedly unsuccessful, a premature automatization process takes place as the working memory capacity is limited and needs to free space to solve other problems. Solms (51) conceptualizes this illegitimate or premature automatization of a procedural pattern as repression. The repressed leads to repetition, as it cannot be reconsolidated. This regular process is compromised in people who suffer from addiction, either before and/or after the onset of the addictive illness. Solms (51) has suggested that addiction is an attempt to skip the work needed to meet needs, namely, an endeavor to generate positive affects without performing the actions required to solve problems. As much as this hypothesis may be the case for some people who suffer from addiction, actual repression and the intention to skip work should be parsed. The question that should be asked regarding this model is why a person would want to skip the work if it is the only way in which a specific need can be canceled (i.e., by a specific action) (52). The hypothesized answer is that they cannot work toward drive solution because of the problems derived from their failed social interactions. The procedural and emotional memory systems have learned a restricted and unsuccessful repertoire of solutions to social needs that keep repeating without proper analyzing of errors. Volkow has showed that social stressors impede developmental connections between the PFC and limbic regions (12). The appropriate development of key structures involved in affect regulation and inhibition is compromised, contributing to enhanced impulsive behaviors that can easily lead to addictive disease. Negative affects that strongly motivate toward attempts to find relief are not regulated because of the PFC limitations that originate in the separation distress trauma context. This configuration worsens when the person uses addictive drugs as the PFC will lose more of its functions as a result to the repeated exposure to drugs (8). Addiction becomes a conflict between protracted negative affects and a weakened PFC emotion regulation function. To better understand this problem, an elaboration of the ongoing discussion about the reward and the SEEKING system seems necessary in the context of the separation distress trauma.

### SEEKING, Pleasure and Rewards: the Frequent Misunderstandings About Addiction

Freud (53) hypothesized that addiction is a compulsive search for pleasure that can be compared to a masturbatory activity. From then until the discovery of the reward system of the brain (18) and to date, addiction has frequently been misunderstood as a hedonic search for pleasurable feelings. People ill with addiction are frequently stigmatized, and it is often considered that they can stop using addictive drugs, but that they refuse to do it. The perspective that addiction is a slow suicide is commonly accompanied by the idea that people who use drugs lie and manipulate those around them. They seem to prefer pleasure against anything else. As much as this description may characterize several people who are ill with addiction, it neglects the many factors that account for the complexity of the disease. The more neurobiological findings related to addiction are available, the more we understand why the syndrome takes these characteristics. People who suffer from addiction need social connectedness and frequently inspire the opposite. Some of the known neurobiological facts that relate to addiction should be used to clarify crucial elements in its understanding, which may contribute to enhance the positive outcomes of treatment strategies. Behavioral, cognitive, neurobiological, symptomatic, and subjective aspects seem to correlate, explaining the diverse features that characterize addiction.

The reward system that Olds & Milner (18) described associated with the self-stimulation of mesolimbic regions. Rats would press a lever to receive electrical stimulation in those regions and, after having experienced it, they compulsively pressed the lever to gain more of that stimulation. They stopped caring for previously rewarding objects, such as food and the company of sexual partners. Hence, Olds & Milner interpreted that their rats were getting a positive reinforcement, i.e., a reward. The chemical stimulation of the mesolimbic pathway has the same effects that Olds & Milner observed for the electrical stimulation. Addictive drugs initially increase the release of dopamine in the mesolimbic reward pathway, particularly in the nucleus accumbens (NAcc) (3, 54). The person who starts using drugs may repeat this behavior as an attempt to look for pleasure.

When the brain has not been exposed to drugs, the ventral tegmental area (VTA) sends constant tonic firing to the neurons of the PFC. When an extraordinary event takes place, phasic firing increases the release of dopamine activating D1 receptors, i.e., the hypothesized requisite to experience a reward. The initial use of addictive drugs results in phasic firing of dopamine, thus interpreted as a reward. The enhanced repetition of drug use eventually ensues a decreased reactivity of dopamine to regular rewards. Using drugs becomes frustrating. However, the expectation of using an addictive drug does increase dopamine release in dependent users, suggesting reduced D2 modulating receptors in people who suffer from addiction (33). Low expression of D2 receptors in the striatum has been associated with impulsivity and enhanced propensity to use drugs compulsively (7) along with dysfunction of the PFC (8). These data show that the brain is modified in addiction. Volkow has proposed that addiction is an acquired brain disease (55) and many treatments have attempted to tackle its neuroadaptations [see (4) for a brief review], yet the complexity of addiction requires attempts at integrating its many dimensions.

It has been suggested that genetic variables explain half of the risk of developing addiction when using drugs (56) with epigenetics playing an important role when environmental risk factors are associated (57). Some factors involved may play a pivotal role in the vulnerability to addiction because of the potential correlates between behavior, neurobiological features, and phenomenological experience.

Several studies have acknowledged that increased activity of the mesolimbic dopamine pathway does not relate directly to rewards [e.g., (16, 33, 57–61)], but it was probably Panksepp (17, 19, 23) who emphasized the need to understand the psychobiological nature of the dopamine mesolimbic system. He expanded the notion to a SEEKING system that would explain that the dopamine mesocortic-mesolimbic pathway does not signal rewards, but the prediction of a reward as a secondary process learning. This system does not generate sensory pleasure, but a psychomotor eagerness to seek for objects to satisfy basic needs and to avoid distress. The SEEKING system is a primary affective system that promotes exploration of the environment to find resources to survive. Its objects are determined by the input of different regions in the brain that indicate what should be looked for, including memory systems and the lateral hypothalamus. Thus, SEEKING is life. No homeostatic or affective need can be met without engaging with the outside world, which is explored, accompanied by the subjective feeling of curiosity. Panksepp underscored that there are several reward systems in the brain, not one. In particular, the SEEKING system looks for rewards and its positive feeling relates to an expectancy of finding satisfying objects that can link to Berridge's idea of a “wanting” system (16). To clarify, there is no reward while SEEKING. Only when the satisfying object is found can pleasure be available, which is mediated not by dopamine, but mainly by the EOS (17, 19, 23) that relates to a hedonic hotspot in the NAcc, namely, the “liking” system (62). Panksepp (19, 23, 27, 63) identified the periaqueductal gray (PAG) as the most important region of the brain to generate emotional feelings, including pleasure.

The distinction of the pleasure found in rewards and the SEEKING of a reward seems to play a crucial role in the vulnerability to addiction and in its course. Most proposals that describe addiction explain its associated behavior, neurobiological implications, and psychiatric characteristics. Few consider the corresponding subjective experience. Addiction is treated as though there were no subject to live through its different stages and symptoms. Panksepp's SEEKING system claims for an affective indicator that is consciousness in itself (30, 49, 64) along with the rest of the basic emotion systems, including the social instincts. A re-interpretation of addiction is then possible.

A voluntary decision to experiment with drugs is frequently explained by feelings of curiosity and/or recreational purposes. The user knows that the drug will change their state of consciousness, which may decrease the inhibition function of the PFC (8, 13) resulting in the brief experience of e.g., relaxing and having fun depending on the type of drug used (22, 40). This modified state of consciousness may cause feelings of pleasure that most likely will be searched again. Any person may experiment with the effects of legal or illegal drugs without it necessarily becoming a problem. If they do not engage in binging, intoxication, and/or too frequent episodes of drug use, the probability that the person will develop an addictive disorder is relatively low. However, epigenetic factors may play a relevant part in the risk of developing addiction, particularly if there is a background of previous affective struggle derived from the two traumatic contexts described as extreme and ambivalent. When that is the case, a negative affective state is constantly present recruiting neurochemical cascades linked to depression, either clinical or latent that come from a problem with the social instincts and their secondary process affective patterns, later resulting in the experience of protracted PANIC/GRIEF activation characterized by increased KOR activity (25, 26). Dynorphin release can shut down the SEEKING system or decrease its activity (25). Depression then represents an enhanced risk of developing addiction (Figure 2).

The negative reinforcement hypothesis (9), also known as the dark side of addiction (38, 39), has been proposed as a powerful source of motivation to use drugs compulsively. Withdrawal causes a negative affective state characterized by the release of corticotropin-releasing factor (CRF) and dynorphin. The user then SEEKs relief from anxious and painful feelings by using the drug. However, this proposal excludes the existence of a negative reinforcement before the use of drugs. When a person has been exposed to the extreme or ambivalent conditions of trauma, there is a previous difficulty in experiencing rewards related to various factors, including those associated with social instincts and SEEKING. This may cause a self-medication attempt (40, 41) that may result in addiction. The negative affective states derived from the repeated use of drugs add to the previous unpleasurable feelings that motivated using the drug initially. As tolerance, craving and abstinence increase in intensity, the PFC is left with fewer resources to stop using drugs, to acknowledge the problem and hence to look for treatment options (13).

In terms of the social instincts, the person exposed to the extreme or ambivalent separation-distress trauma will experience difficulties in their relationships with others. Johnson (65–68) has extensively worked on an opioid-tone model that hypothesizes a correlation between opioid tone and proximity to others. If a person feels lonely, their opioid tone is low and can be modulated by the company of others. Protracted PANIC/GRIEF activations because of separation-distress trauma translate into psychic pain (25) that could cease with appropriate connectedness to others. However, people who experience protracted separation-distress trauma frequently experience problems with trust, fearing to be repeatedly hurt. Avoiding negative feelings is instinctive; using drugs can briefly ease the pain derived from the different formats of separation distress trauma. Specifically, an attempt to modulate opioid tone by neurochemical manipulation may indicate hopelessness and depression. Trauma causes FEAR and RAGE, which, in combination with PANIC/GRIEF, result in enhanced unpleasurable feelings that have an influence one on the other. A neurochemical cascade characterized by the increased release of CRF (25, 27) and simultaneous decreased MOR activity and increased KOR activity, along with other neurochemical neuromodulators is common in depression (23, 25, 26). This neurochemical configuration matches some of the negative reinforcement features described by (38) related to the dark side of addiction. Further investigation of the neurochemical correlates of separation-distress trauma may enhance the chances of designing targeted prevention strategies against addiction.

According to the separation-distress trauma hypothesis, compulsive users of drugs are not predominantly SEEKING pleasure, but relief from negative affective experiences derived from social difficulties. Some of them may engage in using opioid drugs with the goal of directly changing their feelings of loneliness, separation distress and psychic pain, i.e., to target the protracted activation of the PANIC/GRIEF system. Considering that the decision to use certain types of drugs comes from the subjective modifications of conscious states, it is not possible, in many cases, to suppose that users are aware of the neurochemical background state of their nervous system, or of the molecular properties of their preferred drugs. A fundamental hypothesis is that they select them according to the subjective experience that accompanies intoxication. They can be considered “wild psychiatrists” (20) who run spontaneous empirical testing of different drugs, doses, frequencies, and combinations (37) expecting to feel different; this elaborates on Khantzian's self-medication hypothesis (40, 41). The question then is why some users who suffer from separation distress trauma may prefer drugs that directly or indirectly affect the SEEKING system. No simple answer can be attempted.

One speculative and insufficient answer is that a group of drug users fear the effects of opioid drugs and decide not to try them. However, based on the empirical self-medication hypothesis (40, 41), some may try which relates to the social nature of human beings and its association with the opioid-tone model proposed by Johnson (66). The basic instinct to attach is necessary to survive and is partly modulated by the EOS (23). Opioid drugs cause subjective effects that can be split into two main categories. The first one includes pleasure, analgesia and sedation. The second category entails the modulation of the need for proximity to others (66). High opioid tone contributes to the subjective feeling that the person does not need to relate to others as can be seen in Autism Spectrum Disorder (ASD) (65). This feeling may cause the pleasurable idea of self-sufficiency, which would apparently solve the problem of failed trust derived from early childhood separation-distress trauma. Once the effects of the drug expire, the user is reminded of how dependent they are on the drug and on the people that they get it from. The previous problem with relationships persists and the effects of opioid drugs turn into a grievous illusion of alleviating the pain derived from a PANIC/GRIEF protracted activation. The self-locking nature of addiction attempts to eliminate the basic need of attachment, ensuring the persistence of a negative subjective experience that worsens because of the dark side of addiction (38, 39). The user strives to cease unpleasant feelings by using drugs. The negative affect as an indicator of unmet needs (30, 50) does not cease until specific problems are solved. Unpleasant affects become the conscious reminder of our need to take action to solve problems and meet our needs to survive. People who use drugs neurochemically manipulate the indications of negative affects because they have failed at solving their problems. A child who suffers from the extreme or ambivalent contexts that cause chronic depression can hardly change their environment and social context; they cannot trust others and automatize deficient relationship patterns that entail constant frustration of their attachment needs that result, not in repression as suggested by Solms, but in an enhanced conscious state of negative affect that becomes unbearable. What the chemical manipulation of SEEKING brings is not pleasure or reward, it is hope and positive expectation (23). Protracted feelings of depression entail hopelessness, which is associated with decreased SEEKING dopamine activity. The inability to find a solution to meet the basic need of attachment leads to the hypothesis that using addictive drugs can be understood as an attempt to survive. Without increased activity of the SEEKING system, no search of connectedness will take place.

We are then left with the conundrum of why addiction becomes a self-locking illness that gives scarce opportunity to help, or otherwise said, why addiction is an illness against life (10). A part of the social support that people ill with addiction need is that clinicians understand this call for help despite the enhanced difficulties that a self-locking illness poses.

### The Treatment of Addiction: Revisiting the Self-Locking Nature of Addiction

The current models of treatment for addiction have been thoughtfully designed and cover its many dimensions from a multidisciplinary perspective. Yet, known by many clinicians, addiction still represents an enormous challenge as the outcomes of the different models are frequently not sufficient. The self-locking characteristics of addiction emphasize the need for better prevention programs, but that task has also faced defying difficulties. The treatment of addiction has largely benefited from recent neurobiological findings and from experience. It can be asserted that pertinent steps have been adopted. When a model of treatment seems to be good, but the distinctive characteristics of the disease show that more is necessary, looking into details may result helpful. Hence, this section does not intend to offer novel steps to follow, but to provide qualitative content to complement the different formats of treatment. Addiction is an illness guided by affect. A deeper understanding of the role that social instincts play before and during addiction may contribute to how the treatment is conducted.

#### When the Treatment Fails

As previously described, the first difficulty is that people ill with addiction consider the need for treatment. Denying the existence of a problem leads to the idea that no help is needed. Drug-dependent people know that they have problems in several dimensions, including their health and their quality of life (9, 14) but they cannot accept it as it entails a change that seems impossible. Furthermore, repeated use of addictive drugs leads to dysfunction of the orbito-frontal cortex via the striato-thalamo-orbitofrontal circuit involved with drive and compulsive behaviors (13) that add to an impairment of self-awareness (69). Too many factors add up to impede the search for treatment; that is the primary concern about the self-locking nature of addiction.

Social support is considered a major contributor to the treatment of addiction (4), and it may play an important role in the format of an intervention. The person ill with addiction is then aware that they are important to a group of people, which many times becomes a key motivator to accept the problem and the treatment. However, the person may be unconvinced and oppose several steps of their treatment, hence they do not commit. Many drug users feel at risk when they are told that they should stop using addictive substances. Their everyday life may predominantly revolve around planning to use drugs, using them and recovering from their effects (9, 70). The idea of abstinence triggers anxiety coupled with craving and withdrawal, and in many cases with the negative affect that existed before addiction. The person may then abandon the treatment and relapse. A new cycle is necessary in these cases, sometimes repeating several times without success. Few users try again until they succeed. The risk in every attempt is that they may stop looking for help and become convinced that they cannot be treated. They may feel locked in their illness while suffering the negative consequences it involves.

The next challenge for those who stay in treatment is to keep the outcome. Volkow (4) has described addiction as a chronic brain disease which entails a constant effort to avoid relapse. PFC inhibition functions are needed to regulate the persistent craving triggered by many internal and external stimuli. Cognitive-behavioral treatments have proved useful in identifying stimuli and to generate more efficient strategies to cope with them. However, a powerful internal stimulus is the factor that motivated the compulsive drug use, i.e., depression (20, 37). Taking drugs may have helped to relieve temporarily the chronic negative affect related to separation-distress traumas, but the protracted need for a reliable caregiver remains unsolved. Hence, the negative affect endures becoming a relevant threat for relapse. Social support is less likely to be accepted in these cases. This is a major concern as people provide the treatment. If the person ill with addiction cannot trust others, they cannot trust the treatment and resist against it (47).

#### Templates of Social Relationships: the Pathology of Secondary Process Affects

Addiction is a chronic illness that requires long-term treatment. Different modalities address specific aspects of the disease to detox and rehabilitate. Abstinence is one of the primary goals and medications that tackle different symptoms based on neurobiological data are helpful. Withdrawal, craving, anxiety, and sleep disorders are examples of symptoms that can improve with the help of pharmacological agents (4). Nevertheless, the treatment should consider that the person ill with addiction has a subjective experience of the different stages of the disease. Consider the long time it took to acknowledge that neurological patients could benefit from psychotherapy additional to their neurorehabilitation treatment (71). The addicted person is a special case of a neurological patient as their brain has been modified because of the use of drugs and to the damage that they cause. It has taken too long to integrate the neurobiological, behavioral, psychiatric, and subjective aspects of the disease and particularly to treat the feeling experience associated with an acquired brain pathology (55). The problems that people had before using drugs combine with the problems that addiction brings. An instance is the case of anxiety which is present in many drug users. While they initially tried to reduce its intensity, using drugs in fact increases anxiety with the engagement of the brain's stress system that includes the bed nucleus of the stria terminalis, the central medial amygdala, and the posterior shell of the NAcc (3, 33, 38). The many brain modifications that addiction entails determine higher intensity of negative affects.

Addiction begins as an attempt to change a subjective affective state. The modification of the mesolimbic dopamine pathway indicates it is a pathology that involves drives and instincts (17). There is a loss of will that results in repeating the same self-aggressive behaviors that are associated with internal and external stimuli. Repetition enhances the cycle of addiction, locking the person in social isolation, which increases the vulnerability to addiction. Drug users try to stimulate their SEEKING activity because of the loss of hope (20).

The need for others is instinctive. If a person cannot trust people because of separation-distress trauma, they live in the contradiction of needing others while rejecting them. Psychoanalysis has described this impossible type of relationship as narcissistic (42, 72), anaclitic (43, 44), or allergic (73) just to mention a few proposals. The frustration derived from this paradoxical type of relationship leads to enhanced RAGE that becomes a learned pattern of connecting with others and is stored in procedural and emotional memory systems. Psychoanalytic treatments are based on the understanding of the transference and the countertransference. The narcissistic features of the relationship patterns do not benefit from the classical interpretations of psychoanalytic technique (74). To interpret a symptom, a symbol based on a false connection should be present (46). This is not the case of addiction; thus, a proper psychoanalysis is not indicated. However, a neuropsychoanalytic perspective (67, 75) may become valuable to work with the unconscious patterns of relationship that characterize the transference-countertransference relationship.

#### A Neuropsychoanalytic Assessment of Affect and the Social Instincts

The person ill with addiction needs to accept social support in order to be treated. Drugs instigate social isolation and people close to the person may become exhausted after many failed attempts to help. The person locks themselves gradually losing interest in the external world. In brief, that is the impact of protracted PANIC/GRIEF on SEEKING (25). To attempt a reunion with others, SEEKING must be active. The hypothesis is that people who use addictive drugs may develop an illness of the most basic of all basic emotion systems, namely SEEKING. All pro-social emotional instincts depend on SEEKING, i.e., PLAY, CARE and LUST, as well as the need to attach and to avoid separation distress (PANIC/GRIEF) (17). From the subjective point of view, stimulating the SEEKING system means feeling hopeful about solving the protracted separation-distress trauma. An analysis of the different dimensions of affect, that includes the subjective nature of affect, behavioral responses, cognitive ideas about feelings and the neurobiological correlates of affects, can be helpful (45). An extended version of that assessment is proposed here.

Considering that depression originates from the experience of loss, an analysis of the person's relationships with others may be useful in identifying the main primary social instinct involved. The predominant primary process affects that should be involved in different types of relationships against the ones present in depression are drafted in Table 1. A person whose fundamental relationship with the caregiver has adopted one of the two formats of separation-distress traumas may compromise the rest of the social instincts. Panksepp (19) noted that other types of relationships depend on a secure base. As seen in Table 1, when that basic instinct of attachment is not fulfilled, the rest of the social instincts fail as the person SEEKs for the primary caregiver in peers, couple and even in their offspring. Pathological relationships with others can be observed when a predominant instinct is placed in the wrong type of connection. The other social instincts then remain unsatisfied, too. A detailed analysis can help to assess individual cases to identify the quality of the pathology of relationship patterns that belong to procedural emotional memories. These patterns take the form of a repetition compulsion (76) because of the lack of an appropriate repertoire of options to solve social needs. The automatized repeated behavior ensures that no solution is found. As negative affects become chronic, the person becomes more vulnerable to addiction. Analyzing how these features work in each case will provide information to design a personalized treatment.

**Table 1:** Comparison between the ideal predominant social instincts and in depression for the different types of relationships.

**Type of relationship**	**Ideal predominant social instinct**	**In depression**
With primary caregiver	Attachment to avoid PANIC/GRIEF	Attachment to avoid PANIC/GRIEF
With peers	PLAY	Attachment to avoid PANIC/GRIEF
Couple	LUST	Attachment to avoid PANIC/GRIEF
With offspring and helpless others	CARE	Attachment to avoid PANIC/GRIEF

Several psychoanalytic psychotherapies have emphasized the work with the transference and countertransference to examine patterns of social or object relations [e.g., (74, 77)]. Some of them attempt to use interpretation and some other have modified the technique in various ways. However, no matter the technique, the secondary process associations learned by the procedural and emotional memory systems cannot be forgotten (51). From an evolutionary perspective, it could be hypothesized that traumatic experiences are robust associations that serve the purpose of predicting similar circumstances to avoid future analogous experiences. An interpretation cannot break a relevant association of this type, as they are robust to enhance the chances of survival. The problem is that the pattern generalizes to other stimuli, in this case reacting with the same response regardless of the type of relationship (see Table 1). The joy of PLAY, the satisfaction of CARE and the potential establishment of sexual love through LUST are partially deprived, causing a feeling of disappointment and frustration of the different types of relationships.

Treating the pathology of relationship patterns based on an understanding of social instinct deprivation is a challenging task that depends on the general assessment of damage caused by drug use. However, it is here suggested that a technique based on regulating affect with the help of the therapist may provide the person with a conscious experience of what it feels like to be with the therapist in different situations. Following (74, 78), the satisfied need of feeling safe with the therapist may reduce the basic fault or the feelings of an essential depression (79). These techniques do not use the classical psychoanalytic interpretation but feeling the context or environment of the session and to provide the caregiving function that was missing. A neuropsychoanalytic psychotherapy may help to understand affective procedural patterns thoroughly. As those cannot be modified, new patterns can be experienced in a long-term treatment in which the therapist favors the feeling of reliability and CARE. The latter might guide the person to select new relationships or to change old ones based on the experience of a safe and predictable therapeutic relationship. Although tertiary process resources are always helpful, no cognitive thinking is required for this type of learning. The feeling of protracted separation distress is predicted to linger despite this kind of strategy, which also holds the reality principle that should address that the protracted PANIC/GRIEF activation will probably find no resonance in others who are not the primary caregiver. A process of mourning is proposed to cope with the derived frustration and potential protracted reaction of RAGE that would reinforce the depressive behaviors of self-aggression (20).

In sum, all the regular resources used to treat addiction require that the patient trust the team in charge of the treatment. Without this condition, the expected positive outcome of the treatment may be unlikely. On the neurobiological side, recent findings support the development of new molecules that help with craving, withdrawal, and binging behaviors (4). The more detailed the data, the more targets can be tackled. Reducing the intensity of the negative affect associated with those symptoms can support the work of different formats of psychotherapy. Group therapy directly promotes social support in its various formats. CBT helps to keep abstinence and to create strategies to better cope with triggers of drug use. However, a neuropsychoanalytic technique seems ideal for identifying unconscious associative learning, including that of social secondary process emotions. Models that integrate data related to behaviors, symptoms, subjectivity and neurobiological aspects may guide our current efforts to treat addiction to better outcomes (see Table 2).

**Table 2:** A neuropsychoanalytic assessment of the different dimensions of addiction.

**Dimension of addiction**	**Analysis**
Affect	Which subjective state is modified? Which drugs are used to modify affect?
General assessment of brain modifications	Cortical – PFC Subcortical
Cognitive ideas	Report of thoughts
Unconscious patterns of social relations	Repetition compulsion

## Discussion

Using drugs entails the risk of developing addiction. The principle behind trying different substances relates to trying to change an affective state. Affective neuroscience distinguishes the activation of three basic emotion systems that generate unpleasant feelings: FEAR, RAGE and PANIC/GRIEF. These, along with decreased activity of the SEEKING, CARE, LUST and PLAY systems, may represent a depressive syndrome that translates into neurochemical cascades (25) that can be manipulated either by medications or by psychotoxic drugs. Treating addiction represents an enhanced challenge when it is acknowledged that the symptoms of the disease are secondary to a previous compromise to experience regular rewards. People with antecedents of protracted PANIC/GRIEF activation are more likely to develop psychopathology; addiction is an enhanced risk in these cases. Recognizing the subjective experience of addiction may add quality to the already suitable models of treatment. Social support is a crucial aspect for the treatment, therefore its complexity should be better understood. The neurochemical stimulation of the SEEKING system relates to the expectation of a reward, suggesting that addiction is an illness in search of hope against chronic unpleasant feelings that may originate in the experience of separation-distress trauma. SEEKING is the prerequisite to establish connectedness with others. Our social instincts help us survive. The treatment of addiction should consider an integrative model of the several dimensions involved in order to improve the outcome. Addiction means learning to live with a chronic disease that represents an increased difficulty against the regular social conflicts of life. One of the most challenging issues related to addiction is its self-locking nature, that prompts two essential concerns. The first is that no treatment is looked for because of PFC impairments and denial. The second is that separation distress trauma may contribute to social isolation; when the use of drugs causes addiction, the self-locking characteristic of addiction contributes to enhanced social isolation. The treatment relies on social connectedness, starting with intervention strategies to accepting the help of doctors, therapists and peers. Trusting others may become one of the major challenges. Understanding the different instinctive social needs emphasizes the need for an integrative approach that considers neurobiological, symptomatic, behavioral, and subjective aspects. Neuropsychoanalysis is a strong candidate to take on the challenge.

## Data Availability Statement

The original contributions presented in the study are included in the article/supplementary material, further inquiries can be directed to the corresponding author.

## Author Contributions

The author confirms being the sole contributor of this work and has approved it for publication.

## Conflict of Interest

The author declares that the research was conducted in the absence of any commercial or financial relationships that could be construed as a potential conflict of interest.

## Publisher's Note

All claims expressed in this article are solely those of the authors and do not necessarily represent those of their affiliated organizations, or those of the publisher, the editors and the reviewers. Any product that may be evaluated in this article, or claim that may be made by its manufacturer, is not guaranteed or endorsed by the publisher.

## Footnotes

^1^If the reader is familiar with Panksepp's seven basic emotion systems, particularly of the four pro-social circuits, they may want to skip this section that reviews the key characteristics of the social instincts.
